# Dr. Jarvis on Insanity in the Sexes

**Published:** 1851-01

**Authors:** 


*Dr. Jarvis on Insanity in the Sexes.*—We have received an interesting pamphlet on the above subject, being the publication of a paper read before the Association of Medical Superintendents of American Institutions for the insane, at their annual meeting, at Boston, in June, 1850, by E. Jarvis, M. D., of Dorchester, Mass.

Dr. Jarvis has analyzed statistics collected from various sources to determine, 1st, the comparative liability of males and females to insanity, and the causes in males and females; 2d, the comparative curability in the two sexes; and 3d, the mortality of male and female lunatics.

The conclusions arrived at are, that males are more liable to insanity, than females, but this is not a universal fact, in some countries the reverse being true. The males predominate in the asylums of America, England,

Scotland, Ireland and France. The females predominate in the asylums of Belgium, among the people of Norway, and among the paupers of England and Wales.

As respects curability, statistics show a proportion of cures, in most of the countries from which data are obtained, in favor of females. In the United States, however, the preponderance is slightly in favor of males. The mortality appears to be less in females.

The author adduces statistics to show that man is more exposed to, is less frequently cured of, and fails more under the attacks of disorder of the nervous system than females. The reverse of this is perhaps the common impression.

The general inference from the statistical investigations presented by Dr. Jarvis, would appear to be that sex *per se* does not exert a very notable degree of influence in the development, or issue of insanity. Although this result is negative, it is important that it be ascertained, and the author is entitled to credit for his industry in collecting materials bearing on the subject.

